# Impact of surface charge on the motion of light-activated Janus micromotors

**DOI:** 10.1140/epje/s10189-021-00008-x

**Published:** 2021-03-23

**Authors:** Tao Huang, Bergoi Ibarlucea, Anja Caspari, Alla Synytska, Gianaurelio Cuniberti, Joost de Graaf, Larysa Baraban

**Affiliations:** 1 Max Bergmann Center of Biomaterials and Institute for Materials Science, Technische Universität Dresden, 01062 Dresden, Germany; 2 Helmholtz-Zentrum Dresden-Rossendorf e.V., Institute of Radiopharmaceutical Cancer Research, Bautzner Landstrasse 400, 01328 Dresden, Germany; 3 Leibniz-Institut für Polymerforschung Dresden e.V., Hohe Straße 6, 01069 Dresden, Germany; 4 Institute of Physical Chemistry and Polymer Physics, Technische Universität, 01062 Dresden, Germany; 5 Institute for Theoretical Physics, Center for Extreme Matter and Emergent Phenomena, Utrecht University, Princetonplein 5, 3584 CC Utrecht, The Netherlands

## Abstract

**Abstract:**

Control over micromotors’ motion is of high relevance for lab-on-a-chip and biomedical engineering, wherein such particles encounter complex microenvironments. Here, we introduce an efficient way to influence Janus micromotors’ direction of motion and speed by modifying their surface properties and those of their immediate surroundings. We fabricated light-responsive Janus micromotors with positive and negative surface charge, both driven by ionic self-diffusiophoresis. These were used to observe direction-of-motion reversal in proximity to glass substrates for which we varied the surface charge. Quantitative analysis allowed us to extract the dependence of the particle velocity on the surface charge density of the substrate. This constitutes the first quantitative demonstration of the substrate’s surface charge on the motility of the light-activated diffusiophoretic motors in water. We provide qualitative understanding of these observations in terms of osmotic flow along the substrate generated through the ions released by the propulsion mechanism. Our results constitute a crucial step in moving toward practical application of self-phoretic artificial micromotors.

**Graphic abstract:**

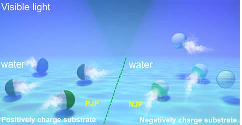

**Supplementary Information:**

The online version supplementary material available at 10.1140/epje/s10189-021-00008-x.

## Introduction

The motion of biological species in nature has inspired scientists from various disciplines to develop a wide range of artificial micromotors [1]. These man-made particles convert chemical or other types of energy (e.g., thermal, electric, magnetic, and acoustic) into mechanical movement [2]. Over the last decade, considerable progress has been made in the design and understanding of micro-and nano-objects that achieve self-propulsion at the microscale [3, 4]. Some of these objects can also perform complex tasks, such as selective loading and cargo transport [5, 6] and drug delivery [7, 8]. They can also serve as elements for biomedical microelectromechanical systems (BioMEMS) [9, 10] and for precision micro-and nanosurgery [11, 12]. Successful application of micromotors to biomedical tasks relies on the motors’ ability to traverse realistic biological environments, which are much more complex than encountered in the typical laboratory setup [11, 12].

In order to realize the diverse applications envisioned, it is essential to achieve control over the temporal and spatial actuation of single micromotors, as well as over their collective behavior. Different methods have recently been developed for controlling the directionality of artificial micro-and nanomotors, as well as regulating their speed and behavior; these include applying external fields [13–16], thermally driven acceleration [17], and chemical stimulation [18, 19]. These routes offer great promise for creating powerful micromachines that can operate independently and meet a variety of future technological needs. However, a less well studied but key piece to the control puzzle is how artificial micro-objects interact with their microenvironment [20–22], both in terms of nearby boundaries [21, 23, 24] and the presence of other (biological) objects [22, 25–27]. The surface properties of both the micromotors and the substrate have been shown to play an important role in determining the motion of self-phoretic micromotors [24, 28–30]. This is to be expected, as their propulsion depends crucially on the local asymmetry in the distribution of the chemical reaction products, the electrical potential, and the fluid flow, all of which can be modified by a nearby boundary. In addition, micromotors have been shown to be attracted to boundaries hydrodynamically [31], and particles possessing negative buoyancy can readily sediment to the bottom. These aspects make such particles more sensitive to the properties of the boundary [24, 31].

Here, we explore the dynamics of the light-activated Janus micromotors, influenced by the electrical surface charges at the boundary and the micromotors themselves. We specially focus on the two types of the Janus particles carrying positive or negative charges at their surface, henceforth labeled as PJPs and NJPs, respectively, for which we demonstrate the opposite direction of their propulsion near the functionalized glass substrate. We classified the coupling between micromotor charge and substrate surface charge on the one hand and the observed speed and direction of motion on the other. Charge density, zeta potentials, and contact angles of the substrate as well as zeta potential of the particles were measured to support the experimental observations. Remarkably, we found no appreciable coupling between the micromotor and the substrate in terms of self-propulsion speed when both were negatively charged, but a significant scaling of speed with the zeta potential of the substrate when both were positively charged. In the case of oppositely charged surfaces, the micromotors became stuck. In this regard, our micromotors’ behavior differs from that recently reported for more commonplace H$_{2}$O$_{2}$-powered micromotors [30], where motion was found to be present for oppositely charged swimmers and substrates. For our motors, we attribute the qualitative change in response for positive and negative like-charged surfaces to the amount of particle-substrate separation, and we explain the observed scaling with surface charge for the former in terms of osmotic coupling. That is the ionic species involved in self-propulsion of our charged micromotor give rise to an osmotic flow along the charged substrate, which influences the perceived speed when the micromotor is sufficiently close. This is conceptually similar to how the speed of a charged colloid is impacted by the presence of a charged wall when the colloid brought into motion by external ionic diffusiophoresis, i.e., by a salt gradient of ions with different mobilities [32]. Lastly, we provide an outlook on the way our results can be exploited in future biomedical applications.

**Fig. 1:**
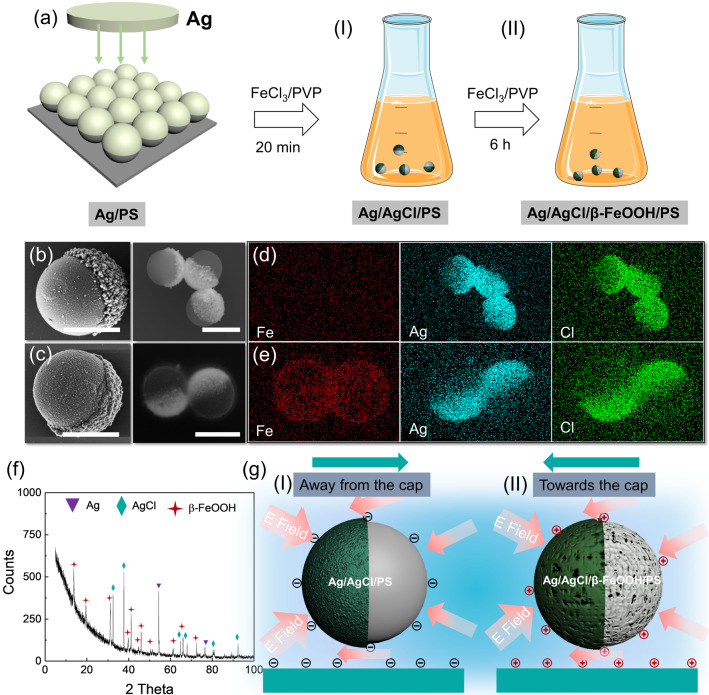
**a** Fabrication process of Ag/AgCl/PS (NJP) and Ag/AgCl/$\beta$-FeOOH/PS (PJP) micromotors (**b**–**c**) Scanning electron microscopy (SEM) images of **b** Ag/AgCl/PS and **c** Ag/AgCl/$\beta$-FeOOH/PS Janus particles. **d**, **e** The corresponding EDX mapping of Fe, Ag and Cl elements. **f** XRD pattern of Ag/AgCl/$\beta$-FeOOH/PS Janus micromotor. The peaks indicated by a red star were identified as $\beta$-FeOOH, International Centre for Diffraction Data (ICDD) PDF database number: 00-034-1266. The peaks indicated by green rhomboid were identified as AgCl (ICDD No:01-071-5209). The peaks indicated by blue triangle were identified as Ag (ICDD No:04-017-4371). **g** Schematic showing the propulsion direction of (I) PS/Ag/AgCl micromotors (moving away from the cap) and (II) Ag/AgCl/ $\beta$-FeOOH/PS Janus (moving toward the cap)

## Results

### The micromotor system

We fabricated two different light-activated Janus micromotors possessing positive and negative surface charges, respectively (Fig. 1). The procedure of Janus-micromotor fabrication follows the recipe of our previous work [33]. A 60-nm film of Ag was deposited onto the self-assembled array of 2 $\mu$m polystyrene microspheres using a thermal evaporation process at a base pressure of $10^{-5}$ mbar. Next, the Ag layer was converted to AgCl in FeCl$_{3}$ solution at room temperature. The synthesis of photocatalysts for two different types of Janus micromotors was realized by controlling the reaction time of the particles with FeCl$_{3}$ solution. Short reaction times (20 mins) resulted in a nanoparticulate AgCl/Ag layer on top of the metalized side of the PS Janus micromotor (Fig. 1a (I)), similar to those demonstrated previously [33, 34]. Further increase in the reaction time up to 6 h led to the hydrolysis of the excessive FeCl$_{3}$ and the formation $\beta$-FeOOH nanocrystals [35, 36], deposited over the entire surface of the Janus particles (Fig. 1a (II)). Additional details on fabrication can be found in the Experimental Methods section.

Figure 1b–c shows the scanning electron microscopy images of both synthesized object types and show substantial differences between particle morphology. The PS side of the first type (Ag/AgCl/PS Janus particles) remained smooth (Fig. 1b). For the second type (Ag/AgCl/$\beta$-FeOOH/PS Janus particles), the whole particle became rough, being covered by $\beta$-FeOOH crystals (Fig. 1c and Fig. S1). This result was further validated using energy-dispersive X-ray spectroscopy (EDX) mapping (Fig. 1d, e). EDX signals from Ag and Cl elements indicated the presence of Ag and AgCl at only one hemisphere for both types of Janus particle. At the same time, the element Fe was homogeneously distributed over the whole surface of the Ag/AgCl/$\beta$-FeOOH/PS Janus particles while being absent for the Ag/AgCl particles. The X-ray diffraction (XRD) measurements further confirmed the presence of the $\beta$-FeOOH phase, i.e., the XRD peaks could be indexed to $\beta$-FeOOH (ICDD card no. 00-034-1266) (Fig. 1f). Finally, $\zeta$-potentials of the particles were measured using Malvern Zetasizer revealing $\zeta _{\mathrm {NJP}} = -15.1 \pm$ 3.6 mV for Ag/AgCl/PS particles (NJP stands for negative Janus particle) and $\zeta _{\mathrm {PJP}}$ = 26.4 ± 3.9 mV for the Ag/AgCl/$\beta$-FeOOH/PS particles (PJP stands for positive Janus particle), see the Experimental Methods section for further details. The sign change in $\zeta$-potential between NJPs and PJPs was due to positively charged $\beta$-FeOOH being homogeneously distributed over the entire surface of the latter ones.

### Substrate functionalization

We used the prepared particles to perform our substrate-interaction studies with functionalized silica substrates; the substrates were covered with covalently bound self-assembled molecular monolayers to tune their $\zeta$-potential. Several negatively charged molecules (Trichloro (1H,1H, 2H,2H-perfluorooctyl) silane (FDTS), succinic anhydride (SA), (3-mercaptopropyl)-trimethoxysilane (MPTS), polyethylene glycol silane (mPEG), Table S1) were chosen to vary the charge on the substrate in a broad range of $\zeta$-potential value ranging from $\sim -30$ to $\sim -60$ mV, respectively (Fig. S3). Additionally, we were able to form a positively charged substrate by functionalizing the plasma-activated silica substrate using ((3-Aminopropyl) triethoxysilane (APTES). Tuning the reaction time from 0.5 h to 6 h led to a range of positive $\zeta$-potential substrates (from $\sim$ 55 mV to $\sim$ 70 mV, pH = 5.6, see Fig. S4), because more APTES was built upon the substrate. Surface density of the involved amino groups at the glass substrate was quantified using UV–Vis spectrometry with the colorimetric method (see and calibration results in Fig. S5). Values of the $\zeta$-potential were obtained via measurements of the streaming current at the silica surface, see the Experimental Methods section for additional details. To complement this analysis, the static contact angle of the substrates was measured in parallel to check for possible correlations (Fig. S6) [30].

### Sensitivity to the illumination

First, we investigated the effect of the particle’s surface charge on their motion by comparing the dynamics of NJPs and PJPs placed on top of like-charged substrates, namely plasma-activated clean silica ($\zeta$-potential $\sim$ -56 mV, Fig. S3) and APTES-functionalized glass ($\zeta$-potential $\sim$ 55 mV), respectively. The motion of a single Ag/AgCl/PS (NJP) was studied in our previous work [33], wherein we found that its self-propulsion is induced by the photocatalytic reaction of Ag/AgCl and that the micromotor’s speed can be tuned by changing the light intensity. Upon illumination with blue (475 nm) light, NJPs were observed to move in the direction away from the cap above a glass substrate, and it further showed a tendency to move far from the boundary (movie 1). In contrast to the NJPs, positively charged Ag/AgCl/$\beta$-FeOOH/PS particles (PJPs) were found moving in the opposite direction, i.e., toward the cap, above APTES-functionalized glass. Importantly, PJPs were also found to stay close to the boundary (movie 1).

**Fig. 2:**
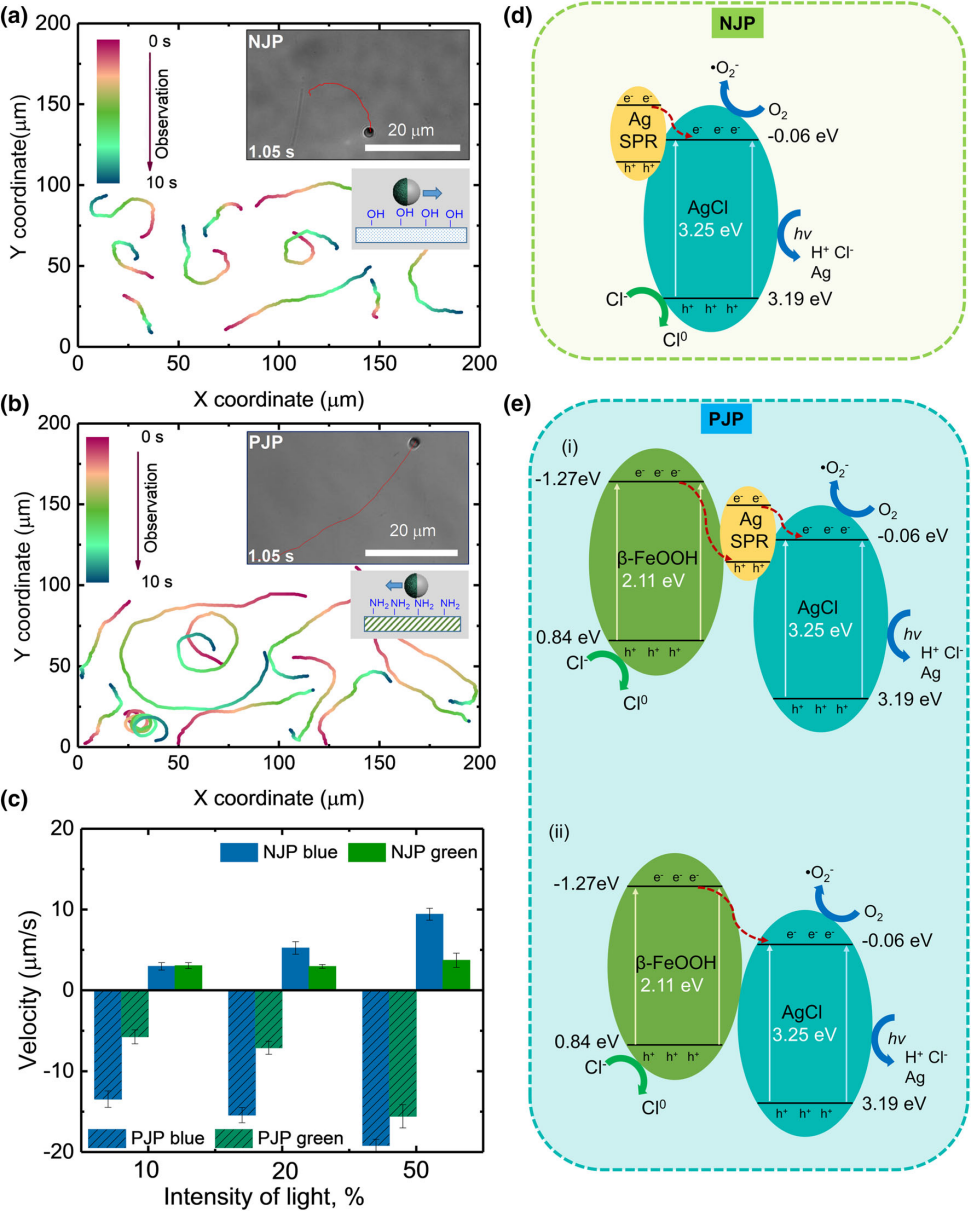
**a** Trajectories of Ag/AgCl/PS (NJP) **b** Trajectories of Ag/AgCl/$\beta$-FeOOH/PS (PJP) under blue light illumination for 10 s. **c** Velocity of Ag/AgCl/$\beta$-FeOOH/PS (PJP) and Ag/AgCl/PS (NJP) motors under blue- and green-light illumination with different light intensities. **d** Proposed mechanism for visible light absorption process of Janus PS/Ag/AgCl (NJP) micromotors based on the surface plasmon resonance (SPR) effect. **e** Proposed mechanism of Ag/AgCl/$\beta$-FeOOH/PS (PJP) under green and blue light illumination

Next, we investigated the motilities of PJPs and NJPs, when these are illuminated using blue (475 nm) and green (555 nm) light with the intensities 10–50% (Fig. S2). Typical trajectories of moving Janus particles are shown in movie 2 and movie 3 and plotted in Fig. 2a, b. Note that a substantial fraction of the particles revealed persistent circular motion. This is due to a torque caused by the imperfect distribution of the photocatalyst over the surface, which leads the inhomogeneity of the reaction across the cap [37]. NJPs and PJPs were both driven by the photocatalytic reactions induced by the two wavelengths (movie 2 and movie 3). In each case, the velocity showed linear increase with the increase in the light intensity in the range from 10 to 50%, see Fig. 2c—blue colored data for blue light and green colored data for green light, respectively. The average speed of PJPs was generally found to be larger than that of NJPs at the same light intensity (Fig. 2c). For instance, micromotors illuminated by blue light reached maximum speeds of 20 $\mu$m/s at 50% illumination intensity, and minimum speeds of 6 $\mu$m/s at a light intensity as low as 10% (10 $\mu$m/s and 3 $\mu$m/s for NJP, respectively). Interestingly, NJPs and PJPs revealed distinct behavior as a function of intensity. On the one hand, the speed of PJPs showed pronounced dependence on the intensity for both blue- and green-light illumination, confirming the efficient light absorption in the broader range of the light spectrum. On the other hand, NJP particles only showed an intensity dependence under blue light, while the change in speed was negligible when varying the green-light intensity. This can be explained by the relatively narrow band absorption of the Ag/AgCl caps that makes NJP micromotors motile only under UV and blue light (Fig. 2d).

The mechanism of motion for Ag/AgCl/PS micromotors was previously discussed in the literature [33, 34, 38], wherein it was shown that the Janus particles are driven by the decomposition of AgCl, which is supported by plasmonic excitations in Ag clusters. The reaction triggers the simultaneous release of protons and Cl$^{-}$ ions. The mobility of the protons is greater than that of the Cl$^{-}$ ions (D$_{\mathrm {H^{+}}}$ = 9.310$^{-5}$ cm$^{2}$ s$^{-1}$ vs D$_{\mathrm {Cl^{-}}}$= 1.410$^{-5}$ cm$^{2}$ s$^{-1}$), which leads to the formation of a compensating electric field. This out-of-equilibrium field is asymmetric around the Janus particle due to the half coating of AgCl (see Fig. 1g) [39]. The field points from the polystyrene part to the cap, and its coupling to the ions in the medium leads to an ionic self-diffusiophoresis of the charged micromotor [34]. The details of the self-propulsion mechanism of Ag/AgCl/$\beta$-FeOOH/PS Janus particles still need to be fully clarified. However, it is reasonable to assume that the driving is similar to that of the Ag/AgCl/PS motors. This insight is already sufficient to explain the observed direction of motion difference between PJPs and NJPs.

The effect of wavelength and the associated difference in response to intensity variation are not fully understood. Under blue-light illumination, the NJPs shows efficient propulsion due to the surface plasmon resonance effect (SPR) of Ag nanoparticles that initiate decomposition of AgCl (Fig. 2d). PJPs were found to move faster than NJPs, which is probably due to the presence of nanocrystalline $\beta$-FeOOH, this is known to be an efficient photocatalyst or co-catalyst in photoreactions, e.g., the photo Fenton process [40]. The nanocrystals of iron oxide-hydroxide are deposited over the whole surface of the particle (Fig. 1c and Fig. S1) and contribute to the absorption of the photons in the visible spectral range [41, 42]. The presence of iron oxide-hydroxide in PJPs generates an additional source of electron–hole pairs under the blue- or green-light illumination: $\beta$-FeOOH serves as a co-catalyst, which provides extra electron to the conduction band of AgCl and accelerates the decomposition of AgCl. The bandgap diagram supporting this scenario of the photocatalytic process at the cap is provided in detail in Fig. 2e (panel (i)). Thus, more ions can be generated in the vicinity of PJP cap; as a result, PJPs reach nearly twice the speed of the NJPs, at the same light intensity.

In contrast, green-light illumination does not lead to a strong plasmonic excitation in Ag clusters. (This is reflected in the weak propulsion and no intensity dependence for NJPs, see Fig. 2a.) This suggests a relatively narrow distribution of Ag nanocluster sizes formed on the cap, implying that Ag cannot be considered as a primary source of electrons that causes AgCl decomposition in the green part of the spectrum. Thus, the $\beta$-FeOOH becomes a crucial catalyst to initiate AgCl decomposition process under green-light illumination, as described in bandgap diagram (ii) of Fig. 2e.

### The effect of substrate surface charge

**Fig. 3:**
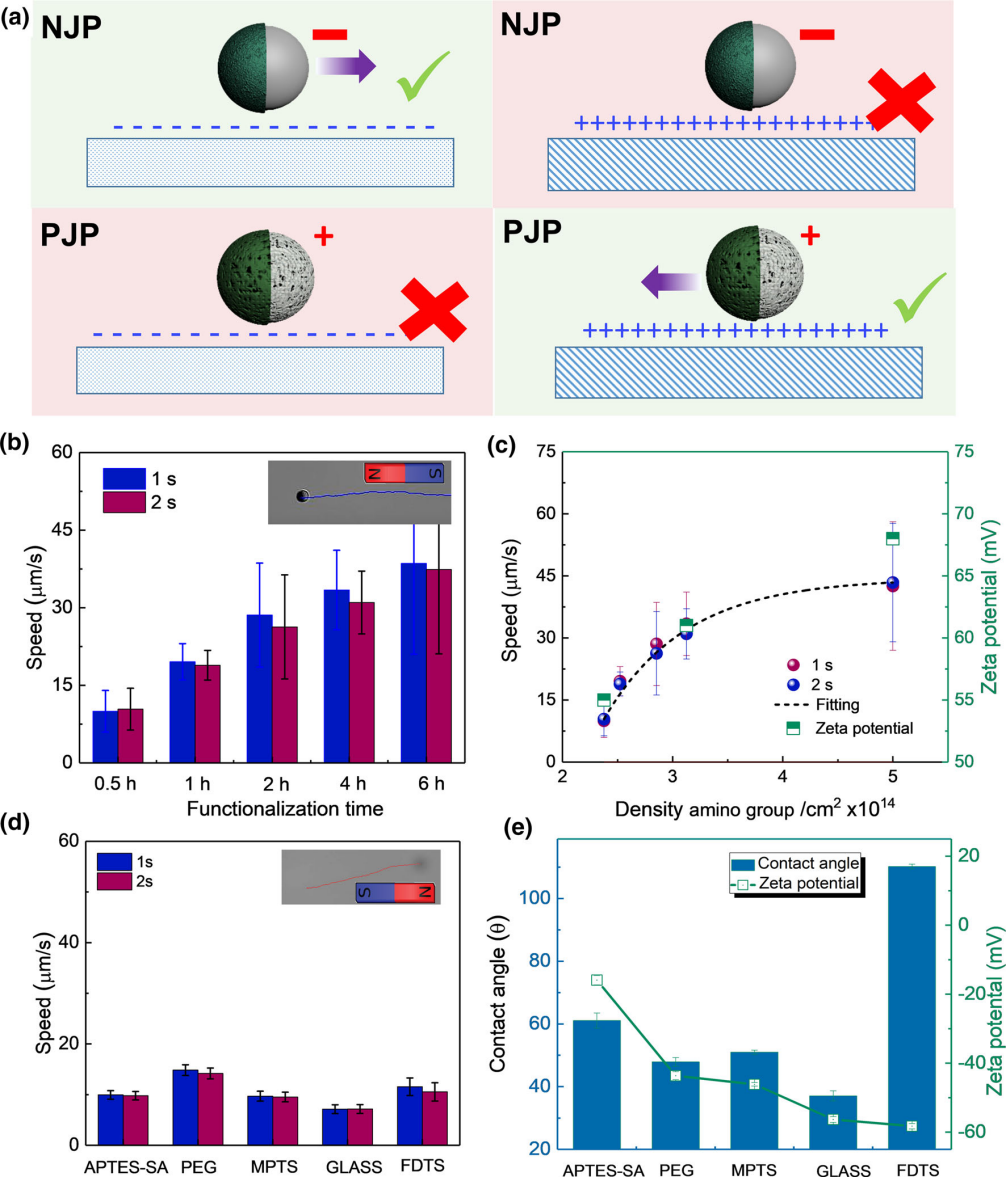
**a** PJP and NJP on positively and negatively charged substrate, respectively. **b** The average speeds of PJP micromotors on positively charged APTES substrates. Error bars are the standard error from ten particles. Insert, the trajectory of magnetic PJP micromotor under a fixed magnetic field. **c** PJP micromotor speeds and substrate zeta potential change as a function of amino group on the substrate. The dashed line serves as the fitting curve. **d** The average speeds of NJP micromotors on different substrates which functionalized with four different molecules. Error bars indicate the standard error from ten particles. The inset shows the trajectory of magnetic NJP micromotor under a fixed magnetic field. **e** Contact angle and Zeta potential values (when the $\hbox {pH} = 5.6$) obtained on different substrates

The surface charge of the substrate may crucially influence the behavior of the micromotors, including their velocity [43, 44]. In order to gain insight into this dependence, we tuned the $\zeta$-potential of the glass surfaces via their surface functionalization (Fig. 3). As a control, we observed that positively charged Janus particles became stuck to a negatively charged boundary and vice versa, see Fig. 3a. Note that in this regard, our system differs from that of H$_{2}$O$_{2}$-powered micromotors, for which there are indications that self-propulsion near an oppositely charged substrate is possible [45].

First, we draw the attention of the reader to the investigation of the motion of PJPs above positively charged substrates. As mentioned above, all PJPs motors moved toward their caps and stayed close to the positively charged substrate under blue-light illumination (movie 4). In this respect, PJPs appeared ideal to investigate the presence of a coupling between the micromotor and the substrate. In order to obtain insight into the velocity of the particles and to remove the interfering rotational fluctuation of Janus micromotors, magnetic multilayers (bottom layers, Co/Pt (0.5 nm/1 nm)$_{10}$) were deposited on top of polystyrene beads to rectify the trajectory of Janus particles using an applied planar magnetic field ($\sim$ 2 mT). We found the average speed of the PJPs to linearly increase with APTES functionalization time (Fig. 3b). Interestingly, this corresponded to a nonlinear increase in velocity with the surface density of the amino groups (Fig. 3c). Zeta potential measurements show fully covered surface already after 2 hours. With longer modification time, we reached nearly stable values (Fig. S4).

Next, we considered the propulsion of the NJPs, which were only observed to move above negatively charged substrates. In contrast to the PJP case, the speeds of NJP micromotors did not show clear dependence on the $\zeta$-potential of the substrate (Fig. 3d, movie 5). The separation distance between substrate and the NJP could explain this phenomenon. Without illumination, i.e., when the NJP is not active, we roughly estimated the substrate-micromotor separation distance to be $\sim$ 1.54 $\mu$m. Additional details about the calculation can be found in supporting information. Upon blue-light illumination, the NJPs displayed a clear negative gravitaxis, i.e., they moved far away from the substrate. The separation distance between the substrate and NJPs showed great fluctuations over time, reaching the separation distance up to $\sim$ 10 $\mu$m (Table S2). The NJPs sedimented back to the substrate once the blue light was switched off.

## Discussion

We will now put the difference between the behavior of PJPs and NJPs above like-charged surfaces into context. Here, it is essential to distinguish between effects that are expected for bulk self-propulsion and those that can be directly attributed to the presence of the substrate. The difference in the direction of self-propulsion between PJPs and NJPs is expected for a driving mechanism based on ionic diffusiophoresis [43]. This does not require coupling to the substrate, as the speed in bulk is directly proportional to the zeta potential of the motor surface to linear order. In absolute terms, the PJPs have nearly double the $\zeta$-potential of the NJPs, this is likely the dominant factor in explaining the observed speed difference between these motors under blue-light illumination in Fig. 2c. Here, we make the reasonable assumption that both the out-of-equilibrium ion fluxes and the electric fields resulting from illumination are not substantially affected by the surface modifications of the particle under otherwise identical circumstances. Effects such as the change in speed as a function of the level of illumination are also predominantly due to bulk behavior. Here, it should be additionally noted that previous research [34] also showed that there was a limited effect of the nearby substrate on the behavior of AgCl-Janus micromotors.

Nonetheless, we did find clear signs of a motor-substrate coupling in terms of speed. Before light illumination, the polystyrene-based micromotors sediment to the bottom of the chamber and therefore are in close proximity to the substrate (the estimated densities of NJPs and PJPs are $\sim$ 1.89 g/cm$^{3}$ and $\sim$ 1.95 g/cm$^{3}$, respectively, both of which are larger than that of water. See the supporting information for additional details on the density calculation and an estimation for the passive height) [24, 31, 46]. This is controlled by a balance between electrostatic repulsion, gravitational force, and buoyancy [47, 48]. However, there are recent indications that this does not necessarily hold for in the active state [45]. Once illuminating the particles with the blue light, we examined our microscopy data and observed that PJPs remained close to a like-charged substrate, while NJPs increased their separation. This is possibly due to the density distribution difference in a single Janus particle, the density of the active cap (AgCl/Ag in our case) is larger than that of water and polystyrene. Thus for a NJP, the cap preferentially orients downward, leading to negative gravitaxis away from the substrate. For PJPs, the $\beta$-FeOOH layer reduces this asymmetry, which might be why these particles remain close to the substrate.

From the above, we surmise that the difference in substrate $\zeta$-potential coupling observed between PJPs (coupling significantly) and NJPs (revealing no appreciable coupling) can be explained by the difference in height above the substrate. Small separations are well-known to affect solute and the electric field distribution around the micromotor, as well as the hydrodynamic flow [22, 26, 49], with the coupling becoming more noticeable the closer the micromotor is to the substrate. Assuming that both PJPs and NJPs are self-propelled via ionic diffusiophoresis, we argue that the coupling to the substrate occurs via the osmotic variant of ionic diffusion. That is, the ionic species released by the decomposition of AgCl also form a gradient along the charged substrate, leading to a fluid flow along with it. This phenomenon is well-known from the work of Anderson [32, 50, 51], who showed that for externally imposed ion gradients speed of a colloid undergoing ionic diffusiophoresis is proportional to the difference in zeta potential between the colloid and the wall: $U \propto (\zeta _{c} - \zeta _{w})$, where $\zeta _{c}$ is the colloid’s $\zeta$-potential and $\zeta _{w}$ is that of the wall. Extrapolating this to the situation of a self-propelled particle, for which we note that the ionic gradients have a substantially different shape, we would still expect a linear dependence of the swim speed on the difference in $\zeta$-potential between the swimmer and wall, to first order. Note that we have access to the surface charge density $\sigma$ $_{w}$ rather than the zeta potential (Fig. 3c), but from the Grahame equation [52, 53] these two are related: $\zeta _{w}\propto {{\,\mathrm{arcsinh}\,}}$ ($\sigma _{w}$). We would therefore expect our speed to scale : $U \propto {{\,\mathrm{arcsinh}\,}}(\sigma )$, with an offset with respect to the origin. This could explain the trend in Fig. 3c, with the linearity of the speed in Fig. 3b, indicating that the zeta potential of the surface changes linearly with the APTES functionalization time. We note that the data in Fig. 3c is suggestive of a zero-speed at a finite APTES surface density, which may be related to a speed inversion when the zeta potential of the swimmer and the surface become equal, through the scaling relation $U \propto (\zeta _{c} - \zeta _{w})$. Lastly, we note that the contact angle does not significantly correlate the speed of our micromotors, which supports a line of argument that is focused on the electrostatic properties of the substrate (Fig. S6).

## Conclusion

We have introduced two types of light-activated Janus micromotors, carrying a positive and negative charge distribution on their surface, respectively, which are driven by ionic self-diffusiophoresis. Using these, we investigated the dependence of the motility on the $\zeta$-potential of the underlying substrate. We showed that the direction of the motion could be inverted by changing the sign of the motor’s zeta potential. The velocity of these micromotors can be further tuned by varying the boundary’s physicochemical properties that they are close to. Motors become stuck on substrates possessing an opposite $\zeta$-potential, but are sensitive to the value of the $\zeta$-potential for like-charged boundaries, provided they are moving sufficiently close to the substrate. We explain this observation in terms of the way the motors self-propel and how this, in turn, impacts their coupling to the substrate. Thus, our study provides insight into the way micromotors interact with their microenvironment, which is a crucial first step toward realizing the ecologically or medically inspired applications.

## Experimental methods

### Materials

(3-Aminopropyl) triethoxysilane (APTES), Polyvidone (PVP), Trichloro (1H,1H,2H,2H- perfluorooctyl) silane (FDTS), Succinic anhydride (SA), (3- mercaptopropyl) trimethoxysilane (MPTS), Dimethyl sulfoxide (DMSO), Boric acid, Iron(III) chloride hexahydrate, Hydrogen peroxide solution (H$_{2}$O$_{2}$) were purchased from Sigma-Aldrich. Polyethylene glycol silane (mPEG, MW 350) was purchased from Nanocs Inc. HFE oil (3M$^{\mathrm {TM}}$ Novec$^{\mathrm {TM}}$ 7500, >99%) was purchased from IOLITEC Ionic Liquids Technologies GmbH. Glass substrates were purchased from VWR.

### Janus particle preparation

Ag/AgCl/PS (NJP) Janus particles were fabricated following the procedure by Wang *et al*. [33] In brief, monolayers of polystyrene (PS) (Sigma-Aldrich cat. no. 78452) spheres with a diameter of 2 $\mu$m were prepared by casting a drop of 100 $\mu$l colloidal suspension (ethanol: H$_{2}$O, v/v 3:1, $\sim$ 2.5 % solids) onto thin glass substrates. Subsequently, we deposited 60-nm silver onto the surface of PS particle monolayers by physical vapor deposition at pressure $\sim$ 7$\times$10$^{-5}$ mbar. Afterward, Ag/PS particles were detached from the substrate using a brush and resuspended in 1 mL deionized water. For the synthesis of the Ag/AgCl layers, Ag/PS particles were further dispersed into a polyvinylpyrrolidone solution (300 mM) and FeCl$_{3}$ (20 mM) mixed solution. The synthesis process was conducted in a dark environment for 20 mins. The resulting Janus particles were washed five times in deionized water and stored in a refrigerator at $\sim$ 4 $^\circ$C until their use. We prepared Ag/AgCl/$\beta$-FeOOH/PS (PJP) Janus particles by reacting an Ag/PS suspension with polyvinylpyrrolidone solution (PVP) (300 mM) and FeCl$_{3}$ (20 mM) by mixing this solution for 1 hour. Afterward, the particles were washed five times in deionized water to remove ferrous ions in solution and resuspend them in 1-mL deionized water. We subsequently mixed suspension with 5-mL PVP (300 mM) and FeCl$_{3}$ (20 mM) solution at 80 $^\circ$C for another 6 hours. During this time, $\beta$-FeOOH was formation on the surface of Janus particle by hydrolyzing FeCl$_{3}$ solution [35]. The resulting Janus particles were washed five times in deionized water and stored in a refrigerator at $\sim$ 4 $^\circ$C until their use.

### Clean glass (activated) substrate preparation

We prepared clean glass by sonicating glass substrates for 5 mins in acetone, followed by 5 mins sonication in ethanol and drying with nitrogen (N$_{2}$). Afterward, these substrates were put it in air plasma for about 5 mins.

### Substrate functionalization

We functionalized our substrates with a variety of chemical groups. The individual procedures are listed below:

*APTES functionalization:* APTES solution was mixed with deionized water and ethanol in a ratio of ethanol:H$_{2}$O:APTES = 100:5:2 and left at room temperature for 10 minutes. Afterward, the activated substrates were immersed into the silanization mixture for a selection of times (30 mins, 1 h, 2 h, 4 h, and 6 h) while being gently shaken. Lastly, the substrates were washed three times with pure ethanol and left in an oven for 15 mins at 120 $^\circ$C.

*FDTS functionalization:* We mixed 100 $\mu$L FDTS with 10-mL HFE oil. We subsequently immersed the activated glass substrates in this solution for 30 mins, also shaking this gently. After functionalization, the surfaces were cleaned two times with HFE and H$_{2}$O. These were then dried with nitrogen and left in the oven at 120 $^\circ$C for 15 mins.

*APTES-SA functionalization:* After the glass substrates were functionalized with APTES, we separately weighed 140 mg succinic anhydride (SA, Sigma-Aldrich) and dissolved it in 1.5 mL DMSO (Sigma-Aldrich). We then mixed in 9-mL borate buffer (200 mM boric acid; pH 8.0 adjusted with NaOH). Next, we incubated the mixed solution with the APTES-coated substrates for one hour at room temperature. Lastly, we rinsed the functionalized substrates with deionized water and dried these with nitrogen.

*MPTS functionalization:* First, the cleaned glass substrates were immersed in a toluene solution of MPTS (1$\times$10$^{-5}$ M) for 12 h. This resulted in a monolayer of mercapto groups. Subsequently, the mercapto-group-modified substrates were treated with a mixed solution of 30% H$_{2}$O$_{2}$–acetic acid (v/v 1 : 5) at 50 $^\circ$C for one hour to oxidize the mercapto groups to sulfonic groups [54]. As a last step, we rinsed the substrates with deionized water and dried these with nitrogen.

*mPEG-silane functionalization:* We prepared a mixture consisting of 10 $\mu$L mPEG, 9.5 mL ethanol, and 500 $\mu$L deionized water. In this mixture, we subsequently immersed the activated glass substrates for one hour, shaking gently during this time. After functionalization, the substrate was cleaned with ethanol, dried with nitrogen, and put it in oven at 120 $^\circ$C for 30 mins.

### Magnetic layer preparation

We made our particles magnetic by depositing multilayer stack of magnetic materials: Pt 5 nm/[Pt 1 nm/Co 0.5 nm]$_{10}$/Pt 5 nm. This was done by using magnetron sputtering at a base pressure of $1\times 10^{-7}$ mbar. Co and Pt were sputtered in an Ar pressure of 810$^{-3}$ mbar at a rate of 0.5 Å/s.

### Electro kinetic (zeta-potential) measurements

Electrophoretic measurement on particles. A Malvern Zetasizer Nano ZSP was used to measure the Zeta Potential of the Janus particles. The sample was dispersed in DI water, and the suspension was allowed to equilibrate for 1 min at 25 $^\circ$C before starting the measurements. For each sample, we performed the measurement three times to obtain standard error.

Streaming current measurement on substrates. Zeta potential was determined with SurPASS 3 (Anton Paar, Graz, A) by streaming current measurements. Two pieces of coated wafer (10 $\times$ 20 mm) were fixed in the adjustable gap cell with double-sided adhesive tape. The cell was mounted at the device equipped with Ag/AgCl electrodes. The gap was adjusted around 100 $\mu$m. The electrolyte solution was KCl (10$^{-3}$ mol/L). The pressure was changed in the range from 600 to 200 mbar. For the calculation, the range 200 to 500 mbar was used. The zeta potential of the samples was measured as a function of the pH value. The measurements started at neutral pH. Two different pairs of wafer were used for the acidic and the alkaline branch. HCl (0.05 mol/L) and KOH (0.025 mol/L) were used for pH-adjustment.

### Contact-angle measurement

The Drop Shape Analyzer—DSA25—was used to analyze the contact angles in this work. Water droplets with volumes 10 $\mu$L were placed on the differently functionalized glass substrates. Lateral video microscopy and integrated software enabled a straightforward analysis of the drop shape. For each substrate, the contact-angle measurement was repeated three times to obtain statistics.

### Tracking and analysis

Janus particles motion was recorded using a 40$\times$ objective (Fig. 2a, b) and a 100$\times$ objective (Fig. 3), both mounted on an inverted microscope. Movies were recorded at 40 frames per second. Fiji was used for particle tracking and obtain the trajectory data [55]. The distance between two adjacent frames was determined using

1 $$\begin{aligned} \varDelta d = \sqrt{ \left( x_{i+1} - x_{i} \right) ^{2} + \left( y_{i+1} - y_{i} \right) ^{2} } \end{aligned}$$

and the time between frames $\varDelta$t = 0.025 s was used to compute the Janus particle’s instantaneous speed $V = \varDelta d/ \varDelta t$. Each particle’s speed was calculated by averaging first 40 frames to obtain statistics. The mean speed (Figs. 2 and 3) was derived by averaging the speed over ten different single particles. The error bars indicate the standard error from this average.

## Supplementary Information

Below is the link to the electronic supplementary material.

Supplementary material 1 (avi 550 KB)

Supplementary material 2 (avi 3018 KB)

Supplementary material 3 (avi 833 KB)

Supplementary material 4 (avi 972 KB)

Supplementary material 5 (avi 955 KB)

Supplementary material 6 (avi 760 KB)

Supplementary material 7 (eml 11 KB)

Supplementary material 8 (docx 1050 KB)

## Data Availability

This manuscript has associated data in a data repository.
