# Supplementary material for: Impact of surface charge on the motion of light-activated Janus micromotors
Source: Eur Phys J E Soft Matter. 2021 Mar 23;44(3):39. doi: 10.1140/epje/s10189-021-00008-x (PMC7987638; doi:10.1140/epje/s10189-021-00008-x)
Supplement: Supplementary file 8 — Supplementary material 8 (docx 1050 KB) [file 10189_2021_8_MOESM8_ESM.docx]

Impact of surface charge on the motion of light-activated Janus micromotors

*Tao Huang^1,2^, Bergoi Ibarlucea^1^, Anja Caspari^3^, Alla Synytska^3,4^, Gianaurelio Cuniberti^1^, Joost de Graaf^5^, Larysa Baraban^2^‬*

^1^Max Bergmann Center of Biomaterials and Institute for Materials Science, Technische Universität Dresden, 01062 Dresden, Germany

^2^Helmholtz-Zentrum Dresden-Rossendorf e.V., Institute of Radiopharmaceutical Cancer Research, Bautzner Landstrasse 400, 01328 Dresden, Germany

^3^Leibniz-Institut für Polymerforschung Dresden e.V., Hohe Straße 6, 01069, Dresden, Germany

^4^Dresden University of Technology, Faculty of Mathematics and Science, Institute of Physical Chemistry and Polymer Physics, 01062, Dresden, Germany.

^5^Institute for Theoretical Physics, Center for Extreme Matter and Emergent Phenomena, Utrecht University, Princetonplein 5, 3584 CC Utrecht, The Netherlands

**1. PJP SEM characterization**


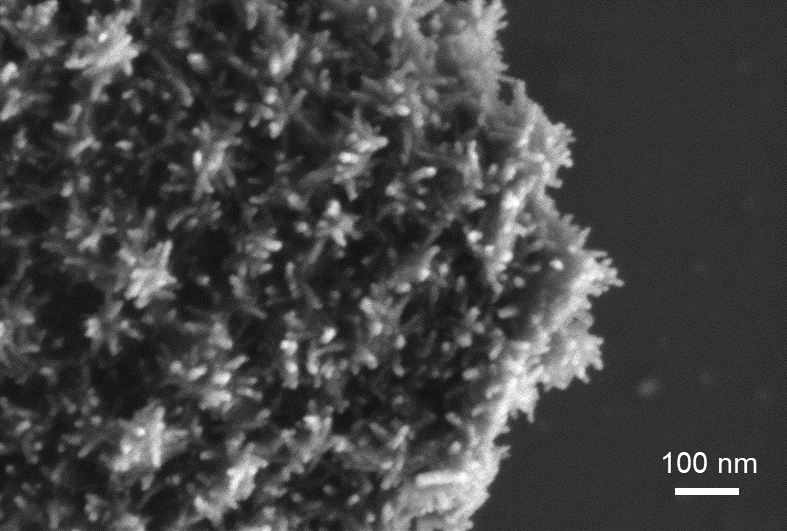


**Fig. S1**. SEM image of PJP Janus micromotors magnified AgCl/Ag coated side of the particle. The scale bar is 100 nm.

**2. Light intensity characterization**


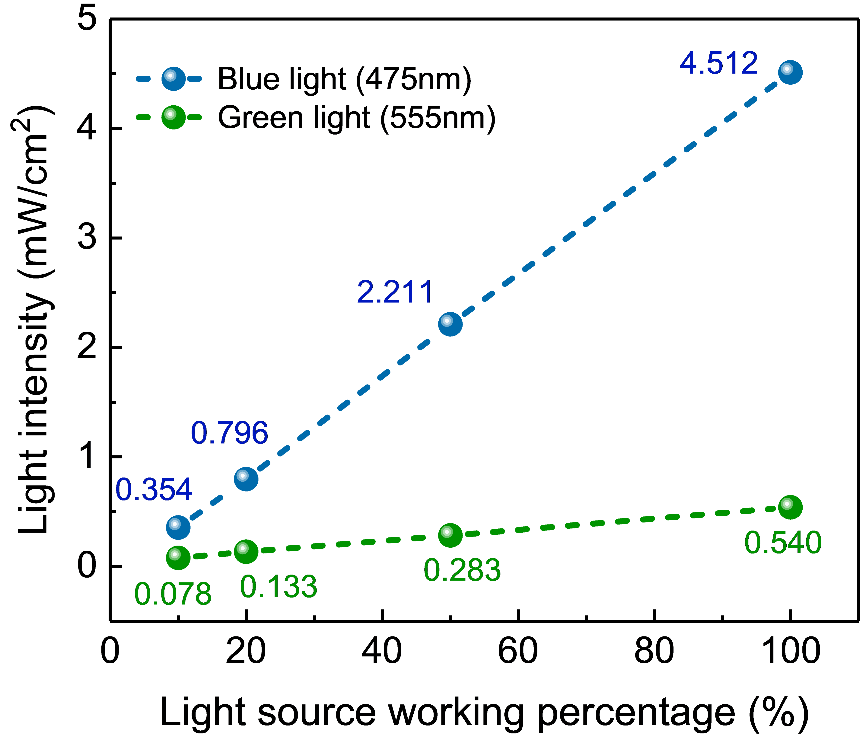


**Fig. S2**. The light source used for driven the micromotor.

**3. Characterization of functionalized substrates**

Table S1. The molecules used for the functionalization.
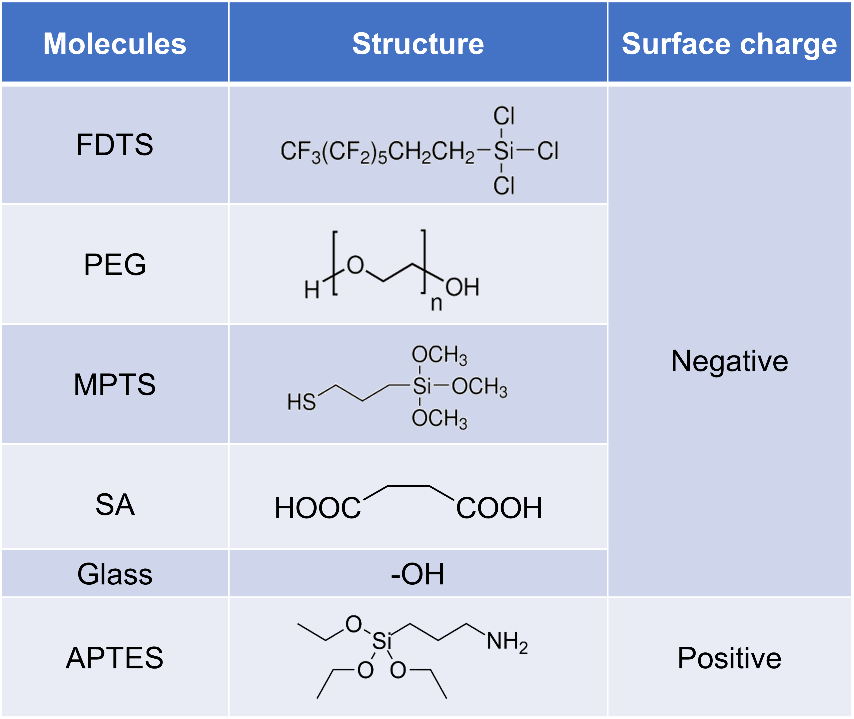


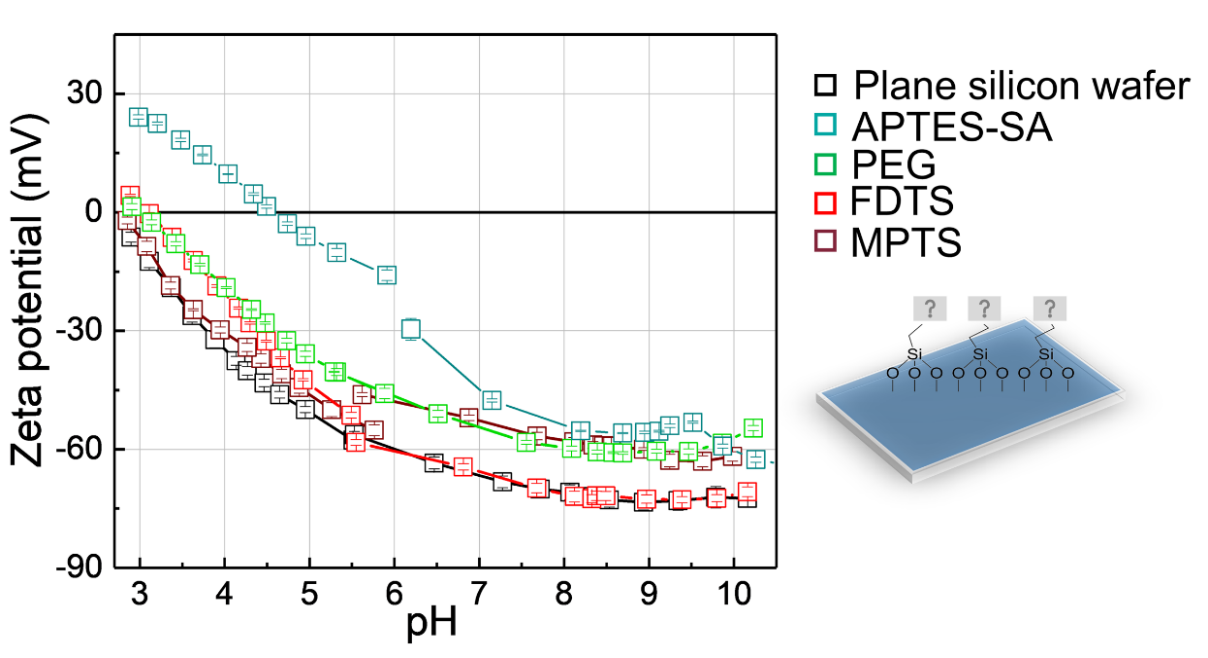


**Fig. S3**. (a) Zeta potential value on differently functionalized silica substrates as a function of pH.


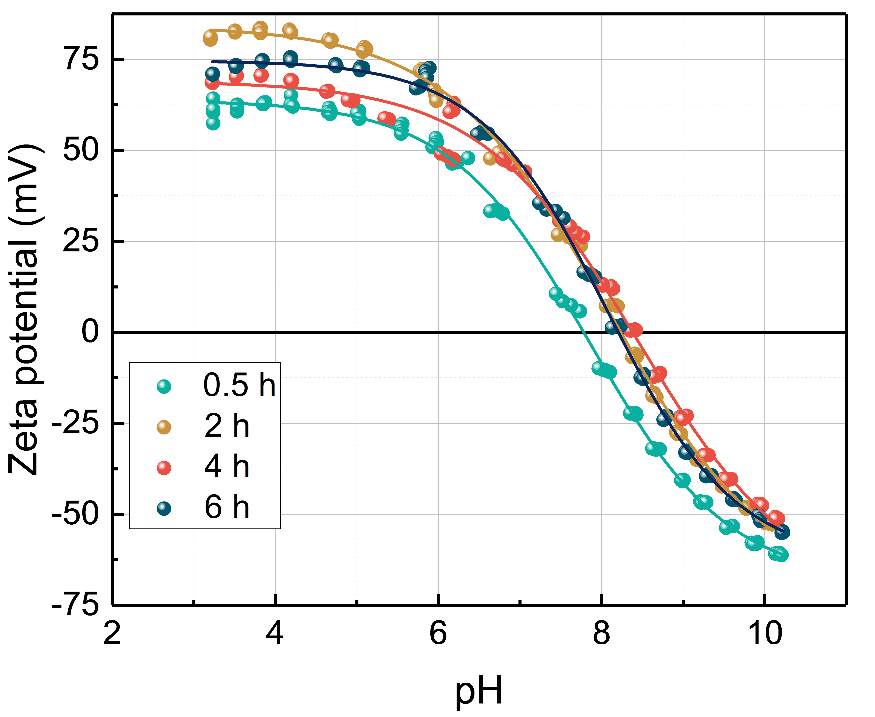


**Fig. S4.** Zeta potential *vs* pH on the APTES-modified substrate with different time of functionalization (green: 0.5 hour, yellow: 2 hours, red: 4 hours, blue: 6 hours).

**3.1. Measure the density of the amino groups on APTES surface**

We use colorimetric methods to quantitatively estimate the density of amino groups on APTES functionalized glass substrates. The experimental steps were as follows: First, The Orange II was dissolved in acidic solution (deionized water adjusted to pH 3 with HCl). Second, the amino-functionalized glass substrates were immersed in Orange II (18 mg/100 mL) solution overnight. The acidic solution ensured that the amino groups at the substrates were protonated. Orange II is negatively charged and will bind to the amino groups with 1:1 molar ratio. After the overnight incubation, the samples were rinsed with acidic solution (pH 3) to remove the unbound Orange II, and dried with N_2_. Then, the colored substrate was immersed in 3 mL alkaline solution (deionized water at pH 10, adjusted by NaOH) for 30 mins to deprotonate the amino groups at the APTES surface and released the Orange II into alkaline solution. Finally, the alkaline solution with desorbed Orange II was further characterized by UV-visible spectrophotometry (shown in Fig. S5c). We could quantify the concentration of Orange II (c__Orange II_) by comparing it with the calibration curve obtained using known concentration solutions. We were able to calculate the total amount of Orange II molecules in the solution (n__Orange II_). The surface area of the substrate was A = 12.5 cm^2^. Therefore, the density of the amino groups could be calculated by d__amino_ = n__Orange II_ /A


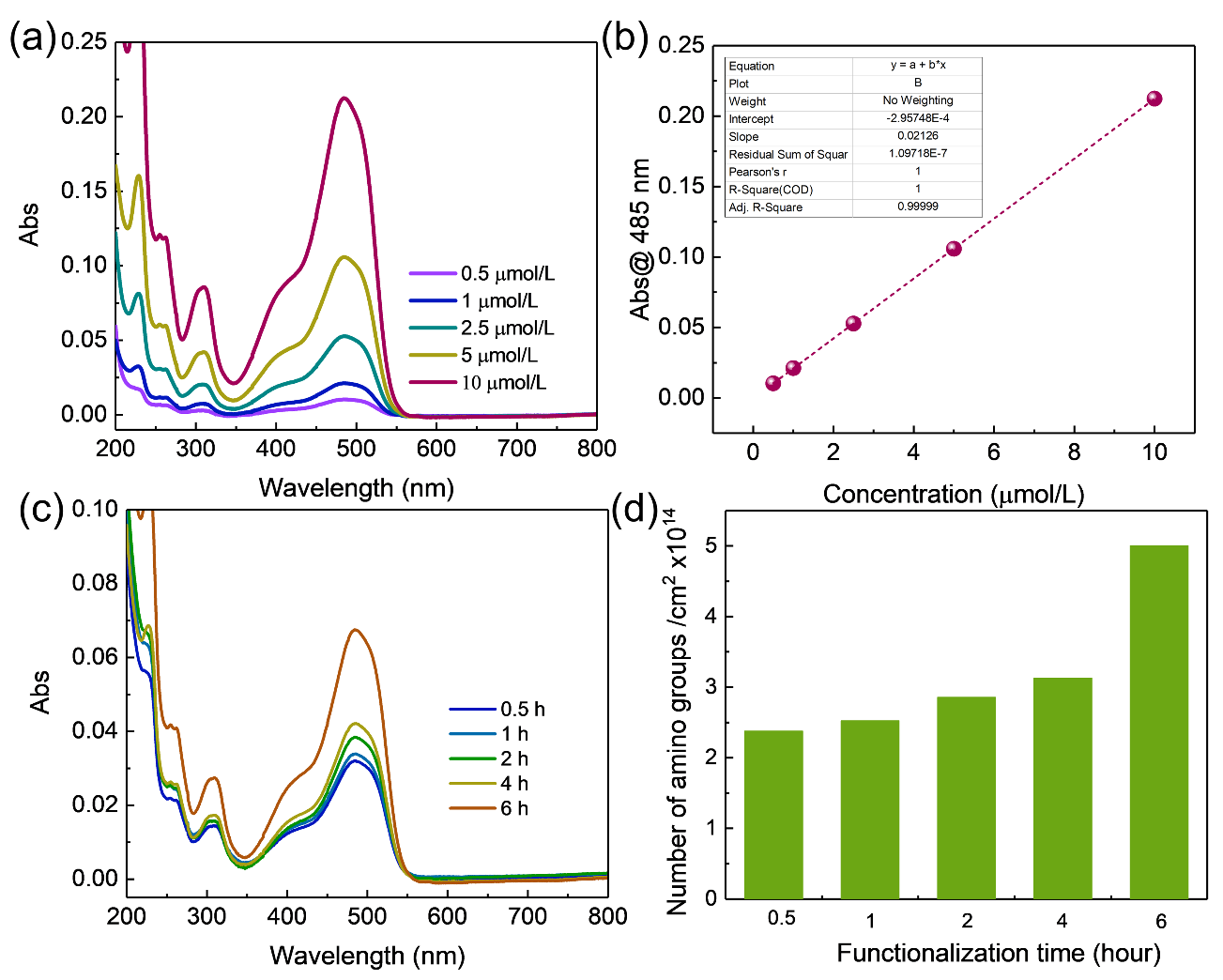


**Fig. S5**. Characterization of the amino-group density. (a) UV-Vis spectra of Orange II at different concentrations and (b) standard curve of Orange II. (c) The UV-Vis spectra of Orange II are released from the substrate. (d) The calculated density of the amino groups on the surface.


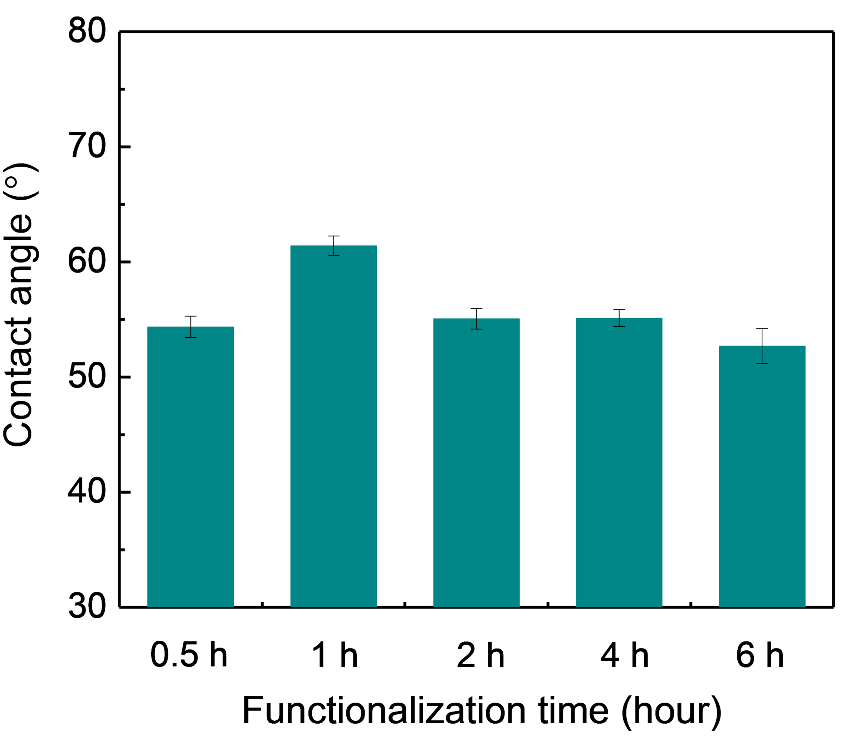


**Fig. S6**. The contact angle of the APTES substrate in degrees as a function of the functionalization time in hours.

4. **Micromotor-substrate separation calculations and measurements**

4.1 Calculation of the micromotor-substrate separation distance

The density of Janus particles is greater than that of water. Therefore, they settle near the bottom of the container, where they assume an equilibrium surface-to-substrate separation. For like-charged particles and substrates, this distance is primarily due to a balance between electrostatic repulsion (modulated by the double layers) and buoyancy. In the following, we compute this balance when there is no activity; activity is known to influence micromotor-wall separation.

The electrostatic repulsion (in the linear regime of Poisson-Boltzmann theory) is given by [1,2]:

$$F_{es}=64\pi\varepsilon\varepsilon_{0}\kappa R\left( \frac{k_{B}T}{e} \right)^{2}\tanh\left( \frac{e\zeta_{\mathrm{wall}}}{4k_{B}T} \right)\tanh\left( \frac{e\zeta_{p}}{4k_{B}T} \right)e^{-\kappa D}$$

Where $D$ is the distance from the surface of the Janus particle to wall, i.e., the separation. $\varepsilon\varepsilon_{0}$ is the dielectric permittivity of the medium ($\varepsilon_{0}$ is that of vacuum), $R$ is the radius of the Janus particle, $\kappa^{-1}$ is the Debye length associated with the electric double layer, and $\zeta_{\mathrm{wall}}\mathrm{and}\zeta_{p}$ are the surface zeta potential of the particle and the wall, respectively. Here, the Debye length $\kappa^{-1}$ is given by [1,3]:

$\kappa^{-1}=\sqrt{\frac{\varepsilon\varepsilon_{0}k_{B}T}{\begin{aligned} \sum c_{i}z_{i}^{2}e^{2} \end{aligned}}}$,

where $k_{B}$ is Boltzmann constant, $T$ is the absolute temperature (here we assume room temperature 293.15 K), and $c_{i}$ and $z_{i}$ are the concentration and number of elementary charges $e$ of an ionic species, respectively.

For our system, we can estimate the relevant parameters in the above equations. We consider the dissociation of carbonic acid as the dominant factor in setting the pH of the solution. That is, for DI water without added salt, the ions in solution come from dissolved $\mathrm{CO}_{2}$, since we did not remove this from the air in conducting our experiments. The equilibrium that balances the concentration of protons and the counterions $\mathrm{HCO}_{3}^{-}$ governed by the following equation:

$\mathrm{CO}_{2\left( \mathrm{aq} \right)}+ H_{2}O_{(l)}=H_{\left( \mathrm{aq} \right)}^{+}$+ $\mathrm{HCO}_{3(aq)}^{-}$ K_a_ = 4.5×10^-7^

where K_a_ is the equilibrium constant. We measured the pH of the solution to be close to 5.65, which agrees well with the pH of water saturated with CO_2_. Therefore, using the above equilibrium, the bulk concentration of protons and bicarbonate ions is 2.24×10^-6^ mol/L. The corresponding Debye length is then approximately 204 nm.

The buoyant force directed toward the substrate is given by the expression:

$$F_{g}=\frac{4}{3}\pi R^{3}(\rho_{p}-\rho_{f})g$$

where $\rho_{p}$and $\rho_{f}$ are the density of the particle and the fluid, respectively, and $g$ is the gravitational acceleration. Balancing these two forces, we find for the separation the following expression:

$$D= \kappa^{-1}\ln\frac{48\varepsilon\varepsilon_{0}\left( \frac{k_{B}T}{e} \right)^{2}\tanh\left( \frac{e\zeta_{\mathrm{wall}}}{4k_{B}T} \right)\tanh\left( \frac{e\zeta_{p}}{4k_{B}T} \right)}{R^{2}(\rho_{p}-\rho_{f})g\kappa^{-1}}$$

To establish $D$ we need to estimate the buoyant mass NJP on negative charged surface. The NJPs were based on PS particles with diameter $d$ = 2 µm; these were half coated with a 60 nm Ag layer, which was subsequently converted AgCl. The overall particle density was calculated using simple geometry and the respective buoyant mass contributions to be around 1.89 g/cm^3^. The Zeta potentials of NJP and the glass substrate were measured to be $\zeta_{\mathrm{NJP}}$ = -15.1 mV and $\zeta_{\mathrm{glass}}$ = -56.3 mV, respectively. This leads to a separation distance between edge of NJPs (Ag/AgCl/PS) and the glass substrate in DI water of around 1.54 µm.

For PJP on a positively charged surface, PJP was synthesized based on d = 2 µm PS particle half coated with 60 nm Ag, and then convert the Ag into AgCl. After reacting this with FeCl_3_ for a long time, a roughly 10 nm thick β-FeOOH had deposited on the surface of the PJP. The PJP's overall density was calculated to be around 1.95 g/cm^3^. The Zeta potential of PJP and the APTES functionalized glass substrate were $\zeta_{\mathrm{PJP}}$ = 26.4 mV and $\zeta_{\mathrm{APTES}}$ = 55 mV, respectively. The separation distance between the edge of PJP and the APTES functionalized glass substrate in DI water was thus calculated to be around 1.50 µm.

4.2. The NJP-substrate separation when there is illumination

We used a Zeiss Axio optical microscope to perform a z-scan of the sample and thereby roughly measure the separation between the Janus particle and substrate. The depth of field (DOF) of our microscope with 100x objective was estimated to be around 500 nm. This means that the microscope can focus to about 1 µm distance in the z-direction, i.e., this method cannot resolve z-direction changes smaller than 1 µm.

The depth of field is given by the expression [4]:

$$d_{\mathrm{DOF}}=\frac{\lambda\times n}{{NA}^{2}}+\frac{n}{M\times NA}\times e$$

Where λ is the wavelength of illuminating light (585 nm), n is the refractive index of the immersion oil (1.515) between the coverslip and objective front lens element, and NA equals the objective numerical aperture (NA = 1.3), The e is the smallest distance that can be resolved by a detector (e = 5.86 µm), M is the magnification (M = 100).

NJPs under blue light illumination shows negative gravitaxis and moving far away from the substrate. In order to estimate NJP-substrate separation distance during light illumination. We first adjusted the microscope and focused on a target Janus particle manually, when the light is off, the Janus particle undergoing Brownian motion, and recorded the z-position of the objective Z__off_. Next, we switched on the light. NJPs moved away from the substrate and out of focus due to negative gravitaxis. We tracked the NJP and brought it in focus again, recording the Z-position of the objective Z__on_ (The separation distance between the substrate and NJPs showed large fluctuations over time, we can not focus on it all the time, only focus on it at a certain moment). This gave us a change in separation by activity Z__on_ – Z__off_, listed in Table S2.

**Table S2.** The change in NJP-substrate separation during light illumination.


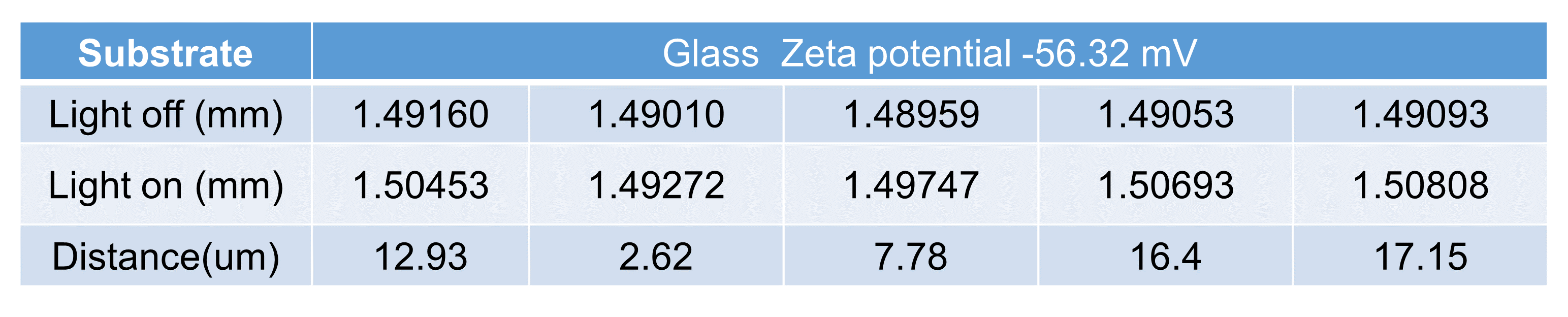


**5. Propulsion speed of NJP micromotor on different substrates.**


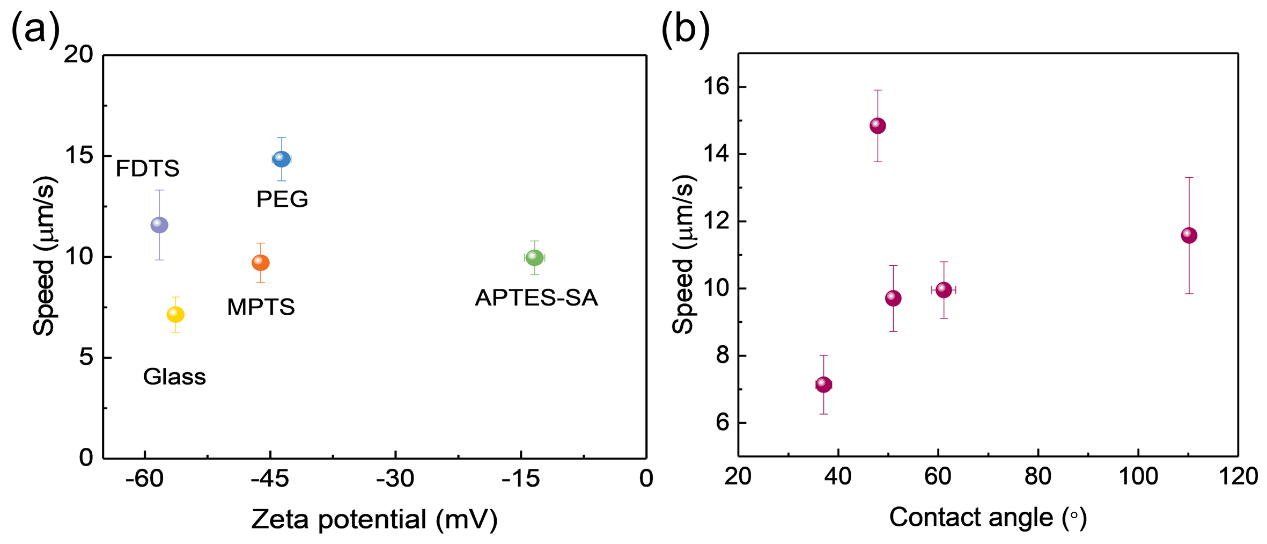


**Fig. S7.** Propulsion speed of NJP micromotor as a function of the (a) Zeta potential in millivolts and (b) contact angle θ in degrees.

**Supplementary movie files**

**Movie 1.** PJPs moving above positively charged surface and NJPs moving above negatively charged surface under blue light illumination.

**Movie 2.** The motion of PJPs moving above a positively charged APTES substrate under blue and green light illumination for different light intensities.

**Movie 3.** The motion of NJPs moving above a negatively charged glass substrate under blue and green light illumination for different light intensities.

**Movie 4.** The motion of PJPs moving above positively charged substrates functionalized with different concentrations of amino groups.

**Movie 5.** The motion of NJPs moving above negatively charged substrates, which functionalized with different molecular groups.

**Movie 6**. Comparison of stuck and Brownian Janus particles when there is no illumination.

1 T.-Y. Chiang and D. Velegol, Langmuir **30**, 10, 2600 (2014).

2 R. W. Verweij, S. Ketzetzi, J. de Graaf, and D. J. Kraft, arXiv preprint arXiv:2009.14733 (2020).

3 T. Li, A. Zhang, G. Shao, M. Wei, B. Guo, G. Zhang, L. Li, and W. Wang, Adv. Funct. Mater. **28**, 25, 1706066 (2018).

4 https://www.microscopyu.com/tutorials/depthoffield
